# Cellular and transcriptomic changes by the supplementation of aged rat serum in human pluripotent stem cell-derived myogenic progenitors

**DOI:** 10.3389/fcell.2024.1481491

**Published:** 2024-10-15

**Authors:** Sin-Ruow Tey, Ryan S. Anderson, Clara H. Yu, Samantha Robertson, Heidi Kletzien, Nadine P. Connor, Kaori Tanaka, Yasuyuki Ohkawa, Masatoshi Suzuki

**Affiliations:** ^1^ Department of Comparative Biosciences, University of Wisconsin-Madison, Madison, WI, United States; ^2^ Department of Surgical Sciences, University of Wisconsin-Madison, Madison, WI, United States; ^3^ Department of Biomedical Engineering, University of Wisconsin-Madison, Madison, WI, United States; ^4^ Department of Surgery, University of Wisconsin School of Medicine and Public Health, Madison, WI, United States; ^5^ Department of Communication Sciences and Disorders, University of Wisconsin-Madison, Madison, WI, United States; ^6^ Division of Transcriptomics, Medical Institute of Bioregulation, Kyushu University, Fukuoka, Japan; ^7^ Stem Cell and Regenerative Medicine Center, University of Wisconsin-Madison, Madison, WI, United States

**Keywords:** human pluripotent stem cells, myogenic progenitors, skeletal muscle aging, F344/BN rat serum, sarcopenia

## Abstract

**Introduction:**

The changing composition of non-cell autonomous circulating factors in blood as humans age is believed to play a role in muscle mass and strength loss. The mechanisms through which these circulating factors act in age-related skeletal muscle changes is not fully understood. In this study, we used human myogenic progenitors derived from human pluripotent stem cells to study non-cell autonomous roles of circulating factors during the process of myogenic differentiation.

**Methods:**

Myogenic progenitors from human embryonic stem cells (ESCs) and induced pluripotent stem cells (iPSCs) were supplemented with serum samples from aged or young Fischer 344 × Brown Norway F1-hybrid rats. The effect of aged or young serum supplementation on myogenic progenitor proliferation, myotube formation capacity, differentiation, and early transcriptomic profiles were analyzed.

**Results:**

We found that aged rat serum supplementation significantly reduced cell proliferation and increased cell death in both ESC- and iPSC-derived myogenic progenitors. Next, we found that the supplementation of aged rat serum inhibited myotube formation and maturation during terminal differentiation from progenitors to skeletal myocytes when compared to the cells treated with young adult rat serum. Lastly, we identified that gene expression profiles were affected following serum supplementation in culture.

**Discussion:**

Together, aged serum supplementation caused cellular and transcriptomic changes in human myogenic progenitors. The current data from our *in vitro* model possibly simulate non-cell autonomous contributions of blood composition to age-related processes in human skeletal muscle.

## 1 Introduction

Age-associated degenerative loss of skeletal muscle mass quality and strength, known as sarcopenia, causes functional decline and loss of independence in seniors (Bruyère et al., 2022; Razaq et al., 2022). Effective treatment strategies are not currently available because the biological mechanisms driving the precipitous decline in muscle mass and function are not well understood. Age-related muscle degeneration is a complex process driven by many age-related factors that are significantly influenced and regulated by the systemic environment.

It has been suggested that circulating factors in blood play a role as non-cell-autonomous modifiers in muscle aging. To support this idea, age-specific effects of sera on skeletal muscle cells have been demonstrated through various experimental designs: young/aged human/murine primary satellite cell culture supplemented with young/aged human/murine sera (Carlson and Faulkner, 1989; Carlson et al., 2009; Barberi et al., 2013; Allen et al., 2021; Carson et al., 2018; Kalampouka et al., 2018), young/aged murine muscles grafted into young/aged murine recipients (Barberi et al., 2013), young/aged murine sera injected into young/aged murine recipients (Sinha et al., 2014; Tripathi et al., 2021), young porcine plasma into aged rats (Enayati et al., 1999), and murine parabiosis studies (Sinha et al., 2014; Carlson and Faulkner, 1989; Conboy et al., 2005). Although rodent models have been extensively used in most studies on muscle degeneration and homeostasis (Conboy et al., 2005; Sinha et al., 2014; Carlson and Faulkner, 1989), there is a limited availability of human skeletal myocytes to study age-associated muscle wasting in humans; such studies have only been done using *ex vivo* myofiber explants or isolated muscle stem cells (Carlson et al., 2009; Barberi et al., 2013; Romagnoli et al., 2021).

Human pluripotent stem cells (hPSCs), which include embryonic stem cells (ESCs) and induced pluripotent stem cells (iPSCs), represent a robust cell source for studying human muscle aging in culture. Recent advancements in the field allow us to prepare myogenic progenitors and skeletal myocytes from hPSCs without genetic modification. These progenitors can be differentiated into mature myotubes upon induction in culture after several weeks (Hosoyama et al., 2014; Jiwlawat et al., 2017). Specifically, our group is preparing myogenic progenitors from hPSCs by a unique culture protocol with free-floating spherical aggregates (Hosoyama et al., 2014; Jiwlawat et al., 2017). Briefly, human ESCs and iPSCs were expanded in medium supplemented with high concentrations of fibroblast growth factor-2 (FGF-2) and epidermal growth factor (EGF). The culture then formed spherical aggregates called EZ spheres. We confirmed myogenic progenitor markers such as Pax3 and Pax7 were expressed in the EZ spheres (Hosoyama et al., 2014; Jiwlawat et al., 2017). Immunocytochemistry revealed that approximately 40%–60% of total cells were positive with Pax7. Furthermore, well-differentiated myosin heavy chain-positive (MyHC^+^) myotubes were identified following sphere dissociation and terminal differentiation (Hosoyama et al., 2014; Jiwlawat et al., 2017).

To understand the relationship between host environment and muscle cell behaviors during the aging process, we sought to determine how circulating blood components would change cellular and molecular signatures in myogenic progenitors and skeletal myocytes. Specifically, we treated hPSC-derived myogenic progenitors with pooled sera from male Fischer 344 x Brown Norway F1-hybrid (F344/BN) rats of 6–9 months or 30–32 months, representative of young adult and aged circulating environments respectively. These F344/BN rats have been known to closely mirror human in terms of timing of late-life, comorbidities of aging, age-related body composition changes and declining performance (Carter et al., 2012). The F344/BN rat strain is a genetically defined rodent model developed, supplied, and recommended by the National Institute on Aging (Sprott, 1991; Sprott and Ramirez, 1997). These rats exhibit a prolonged lifespan relative to the Fischer 344 aging rat model, low detrimental pathologies, delayed onset of typical age-associated symptoms, and relevant age-related declines in skeletal muscle function because this strain lives long enough to experience significant declines in muscle mass (Lushaj et al., 2008; Rice et al., 2005). Additionally, these rats have a small size but still allow us to collect a larger volume of blood samples per animal when compared to similar mouse models.

In this study, we report that the supplementation of aged rat sera to pre-differentiated myogenic progenitors decreased expansion, diminished myotube formation, and slowed maturation when compared to the cells supplemented with young adult rat sera. We also performed transcriptome analysis and determined how the gene expression profiles were affected in human myogenic progenitors following aged serum supplementation in culture. Our current findings indicate that aged blood composition influences the ability of self-renewal and differentiation in myogenic progenitors in a non-cell-autonomous manner.

## 2 Materials and methods

### 2.1 Preparation of rat serum samples

Rat serum samples were collected from male Fischer 344 x Brown Norway F1-hybrid (F344/BN) rats. The procedure was performed in accordance with the NIH Guide for Care and Use of Laboratory Animals, 8th Edition, 2011. The animal care and use protocol was approved by University of Wisconsin School of Medicine and Public Health Animal Care and Use Committee.

Young adult (6–9 months old, 18 rats) and aged (30–32 months old, 18 rats) F344/BM male rats were obtained from the National Institute on Aging colony (Harlan Laboratories, Indianapolis, IN). The F344/BN rat median life span expectancy is approximately 36 months (Turturro et al., 1999). Rats were housed in standard polycarbonate cages in pairs in a light-controlled environment with a 12:12-h light-dark reversed light cycle with food and water provided *ad libitum*. Rats were anesthetized with 4% isoflurane and blood was collected through cardiac puncture using a 21-gauge needle connected to a 20 mL syringe inserted into the ventricle of the heart. Collected blood was transferred into an ice-cold 15 mL conical tube and stored at 4°C overnight to allow the blood to clot. The clot was removed by centrifuging at 1,200 rpm for 5 min at room temperature. Serum was filtered through a syringe filter with a 45 µm pore size (229,774; Celltreat Scientific Products, Pepperell, MA) and stored in a sterile 15 mL conical tube at −80°C. Per age group, serum samples from 18 rats were separated into 3 groups of 6 for the preparation of 3 pooled sera samples. Equal amounts from each of the 6 serum samples were pooled, aliquoted into 1 mL microcentrifuge tubes, and stored at −20°C until needed. For experiments that were repeated 2–3 times, we used a different pooled sera sample each time to increase the reliability of our findings.

### 2.2 Human pluripotent stem cells (hPSCs)

Human ESC line WA09 (H9) and human iPSC line IMR-90 were obtained from WiCell (Madison, WI). Colonies of these lines were maintained using a feeder-free protocol (Ludwig et al., 2006) in mTeSR1 (WiCell) medium on a 6-well plate coated with Matrigel (BD Bioscience; San Jose, CA), and passaged using Versene (Life Technologies, Grans Island, NY).

### 2.3 Derivation of myogenic progenitors from hPSCs

Myogenic progenitors and myotubes were derived from hPSCs using our protocol as previously reported (Hosoyama et al., 2014; Jiwlawat et al., 2017; Jiwlawat et al., 2019; Tey et al., 2022). Briefly, hPSC colonies were manually lifted using a scraper and transferred into culture flasks precoated with poly (2-hydroxyethyl methacrylate) (poly-HEMA; Sigma-Aldrich, St. Louis, MO) to prevent cell attachment to the surface. The colonies were maintained in serum-free expansion medium consisting of Stemline medium (S-3194, Sigma-Aldrich), 100 ng/mL human fibroblast growth factor-2 (FGF-2; WiCell), 100 ng/mL human epidermal growth factor (EGF; Millipore, Billerica, MA), 5 ng/mL heparin sulfate (Sigma-Aldrich), and penicillin/streptomycin/amphotericin B (PSA, 1%v/v; Life Technologies). The colonies formed free-floating spherical aggregates known as EZ spheres within 1 week (Hosoyama et al., 2014; Jiwlawat et al., 2017; Jiwlawat et al., 2019; Tey et al., 2022). The spheres were passaged weekly by mechanical chopping using a McIlwain tissue chopper (Mickle Laboratory Engineering, Surrey, United Kingdom) to allow the maintenance of cell-to-cell contact of spherical cultures for rapid and stable expansion without dissociation to a single cell suspension (Svendsen et al., 1998; Ebert et al., 2013; Shelley et al., 2014).

### 2.4 Sphere growth measurement and calculation

The measurement of sphere growth was performed as we had described previously (Wright et al., 2003; Tey et al., 2022). After at least 6 passages by mechanical chopping, hPSC-derived spheres with a similar size (∼20 um diameter, 32 spheres total) were transferred into a low-attachment 96-well plate precoated with poly-HEMA. Each well contained one sphere in serum-free expansion medium (Stemline medium supplemented with 100 ng/mL FGF-2, 100 ng/mL EGF, 5 mg/mL heparin sulfate, and 1%v/v PSA). Three days later, at least 6 spheres with similar size increase (∼150%) were selected for each treatment group (Day 0). The conditioned medium was fully replaced with fresh expansion medium additionally supplemented with either 10% young adult rat sera, 20% young adult rat sera, 10% aged rat sera, or 20% aged rat sera. On Day 3 and 6, spheres were fed by replacing half of the conditioned medium with the fresh medium. The sphere diameters were measured using the eyepiece reticle of a Nikon Eclipse TS100 inverted microscope (Tokyo, Japan) and sphere volume was calculated as recently described (Tey et al., 2022). Growth rates were determined by normalizing each sphere’s changes in volume relative to the initial volume on Day 0. To verify the reliability of the results, we repeated 3 independent experiments.

### 2.5 Serum supplementation during terminal differentiation

HPSC-derived EZ spheres were dissociated using trypsin (TrypLE, Life Technologies) and then disaggregated in terminal differentiation medium [TDM; Dulbecco’s Modified Eagle’s Medium Glutamax (DMEM, Sigma-Aldrich) containing 2% B27 serum-free supplement (Life Technologies) and 2% PSA]. Disaggregated cells were passed through a cell strainer with 40 µm mesh pore size (Corning Inc., Corning, NY), and plated at a density of 4,000 cells/μL on glass coverslips coated sequentially with poly-L-lysine (0.1 mg/mL) and laminin (50 μg/mL) (both Sigma-Aldrich) (Hosoyama et al., 2014). The plated cells were then cultured in DMEM containing 2% PSA and different concentrations (10% or 20%) of young or aged rat samples. The cells were then maintained in 24-well plates for 1 week or 2 weeks to differentiate into myotubes. For the experiments to study the restoration by young rat serum supplementation (as summarized in Figure 5), the plated cells were cultured for 1 week in DMEM containing 2% PSA with the addition of 2% B27 supplement (control), 20% young adult rat sera or 20% aged rat sera, then cultured for another week with the same or a different treatment. Cells were fed every other day by replacing half of the conditioned medium with fresh medium.

### 2.6 Immunocytochemistry

Immunocytochemistry for PAX7, myogenin (MyoG), myosin heavy chain (MyHC), and titin was performed as described previously (Jiwlawat et al., 2017). For co-staining with PAX7 and MyoG, cells plated on coverslips were cultured for 2 days in DMEM containing rat sera and fixed with 4% paraformaldehyde (PFA) in PBS. The cells were then permeabilized and blocked with 0.1% Triton X-100 and normal donkey serum. After rinsing with PBS, the cells were incubated with mouse monoclonal antibodies against PAX7 [1:40; Developmental Studies Hybridoma Bank (DSHB), Iowa City, IA] and rabbit polyclonal antibodies against MyoG (1:400; Santa Cruz, Dallas, TX) at 4°C overnight. For MyHC and titin staining, cells were fixed with ice-cold methanol, then incubated with mouse antibodies against MyHC (MF20, 1:40; DSHB) or titin (9D10, 1:40; DSHB) for an hour at room temperature. After incubation with the primary antibodies, the cells were rinsed with PBS, then incubated with secondary antibodies conjugated with Alexa Fluor 488 or Cy3 (1:1,000; anti-mouse IgG antibodies, Jackson ImmunoResearch Laboratories, West Grove, PA) for 30 min at room temperature in dark. Lastly, cell nuclei were stained with Hoechst 33,258 (0.5 μg/mL in PBS, Sigma-Aldrich). Coverslips were mounted on slides using a mounting medium (Fluoromount-G; SouthernBiotech, Birmingham, AL).

For Ki67 and Caspase-3 staining, cells were fixed with 4% PFA, permeabilized, and incubated with rabbit monoclonal antibodies against Ki67 (1:100; NB600-1252, Novus Biologicals, Centennial, CO) or rabbit polyclonal antibodies against Caspase-3 (1:250; G748A, Promega, Madison, WI) at 4°C overnight. The primary antibodies were labeled with secondary antibodies and Hoechst, then coverslips were mounted on slides, as described above.

### 2.7 Microscopy and image quantification

Immunocytochemical images were acquired using a Nikon Eclipse 80i fluorescence microscope and a DS-QiIMC charge-coupled device camera (Nikon). Cell counts were performed with NIS-Elements imaging software (Nikon) or ImageJ. Expression levels of PAX7, MyoG, Ki67, and Caspase-3 were calculated as the average percentage of positive cells per total cells represented by Hoechst-positive cell nuclei. The number of MyHC-positive (MyHC^+^) cells was determined as the average percentage of Hoechst-positive nuclei surrounded by MyHC^+^ myotubes (% MyHC^+^) for each field of view. Myotube diameter was measured and presented as the average diameter of all myotubes per image field. For each staining, the graphed values were determined from at least 6 microscopic fields from each group per experiment. To verify the reliability of our findings, we repeated 2–4 independent experiments for cell counting.

### 2.8 Western blot

Treated cells were lysed in radioimmunoprecipitation assay buffer (EMD Millipore, Burlington, MA) with a protease inhibitor cocktail (Thermo Fisher Scientific, Waltham, MA) and 5 mM ethylenediaminetetraacetic acid (EDTA; Thermo Fisher Scientific). Protein concentrations were determined using the DC Protein Assay kit (Bio-Rad, Hercules, CA). Proteins (20 μg per lane) were run on a polyacrylamide gel and transferred onto a PVDF membrane (EMD Millipore). The membrane was immunoblotted with anti-human cyclin B1 antibody (1:1000; 4,138, Cell Signaling Technology, Danvers, MA) and Cell Cycle WB Cocktail (1:250; ab136810, Abcam, Cambridge, MA) containing primary antibodies targeting phospho-cdk2 Tyr15 and beta-actin, followed by secondary antibody conjugated with horseradish peroxidase (anti-rabbit IgG HRP; Promega, Madison, WI). Enhanced chemiluminescence substrate (Pierce Biotechnology, Waltham, MA) was used to detect target proteins on the immunoblot for chemiluminescence imaging using UVP ChemStudio PLUS Imaging System (Analytik Jena US LLC, Upland, CA).

### 2.9 RNA sequencing

Total RNA was isolated from plated down EZ sphere cells that were maintained in the serum-supplemented medium (aged, young, or serum-free) for 48 h using Direct-zol RNA Kit (Zymo Research, Irvine, CA, United States) according to the manufacturer’s instructions. Bulk RNA barcoding and sequencing (BRB-seq) was carried out as described previously with a few modifications (Alpern et al., 2019). Library cDNA preparation was carried out using reverse transcriptase primers for CEL-Seq2 (Hashimshony et al., 2016), as has been described previously (Miyawaki-Kuwakado et al., 2021) in that a barcoded oligo-dT based primer was used for single-stranded synthesis and Second Strand Synthesis Module (NEB, #E6111) was used for double-stranded cDNA synthesis. In-house MEDS-B Tn5 transposase (Picelli et al., 2014; Sato et al., 2019) was used for tagmentation and amplified by 10 cycles of PCR using Phusion High-Fidelity DNA Polymerase (Thermo Fisher Scientific, M0530). The quality of the cDNA was assessed with High Sensitivity D5000 ScreenTape (Agilent Technologies, Santa Clara, CA, 50675592) prior to sequencing. Sequencing of the libraries was carried out using a NovaSeq6000 (Illumina, San Diego, CA). Nineteen cycles were performed for read 1, capturing the barcode and UMI, and eighty-one cycles were performed for read 2, capturing the cDNA insert.

RNA-sequencing analysis was carried out as has been described previously (Miyawaki-Kuwakado et al., 2021). Briefly, sample barcodes and UMIs were extracted using the UMI-tools (version 1.1.4) command umi_tools extract -I r1.fastq--read2-in = r2.fastq--bc-pattern = NNNNNNNNNNCCCCCCCCC--read2-stdout. Barcode extraction was followed by read trimming using Trim Galore! (version 0.6.10) with the -a GATCGTCGGACT option. Read quality was assessed using FastQC (version 0.12.1). After read quality was confirmed, they were aligned to the human genome (GRCh38, grch38_snp_tran) using HISAT2 (version 2.2.1). After alignment, reads were sorted and indexed using SAMtools (version 1.10). The read counts for each gene were generated using featureCounts (version 2.0.6) and the UMI-tools command, umi_tools count–method = directional–per-gene–per-cel–gene-tag = XT. Differential gene expression analysis was carried out using DESeq2 (version 1.40.2) in R (version 4.3.3). Genes with a Benjamini–Hochberg-corrected Wald test adjusted *p*-value < 0.1 and |log2foldchange| > 1 were considered significantly differentially expressed. Regularized variance-stabilizing transformed counts were used to perform principal components analysis (PCA) and generate heatmaps for myogenesis related genes. The clusterProfiler software package enrichGO was used to perform gene ontology (GO) enrichment analysis.

### 2.10 Statistical analysis

GraphPad Prism 9.4.0 (GraphPad Software, Inc., La Jolla, CA) was used to perform statistical analyses. Quantitative data were graphed as means ± standard error of the mean (SEM) from at least two independent experiments. Paired or unpaired two-tailed Student’s t-test was performed to compare two groups. Two-way analysis of variance (ANOVA) was calculated to compare multiple groups with terms in the model for treatment and timepoints, followed up with Tukey’s *post hoc* test. Differences were considered significant when *p* < 0.05.

## 3 Results

### 3.1 Aged rat sera reduced the expansion rate of hPSC-derived progenitor cells

**FIGURE 1:**
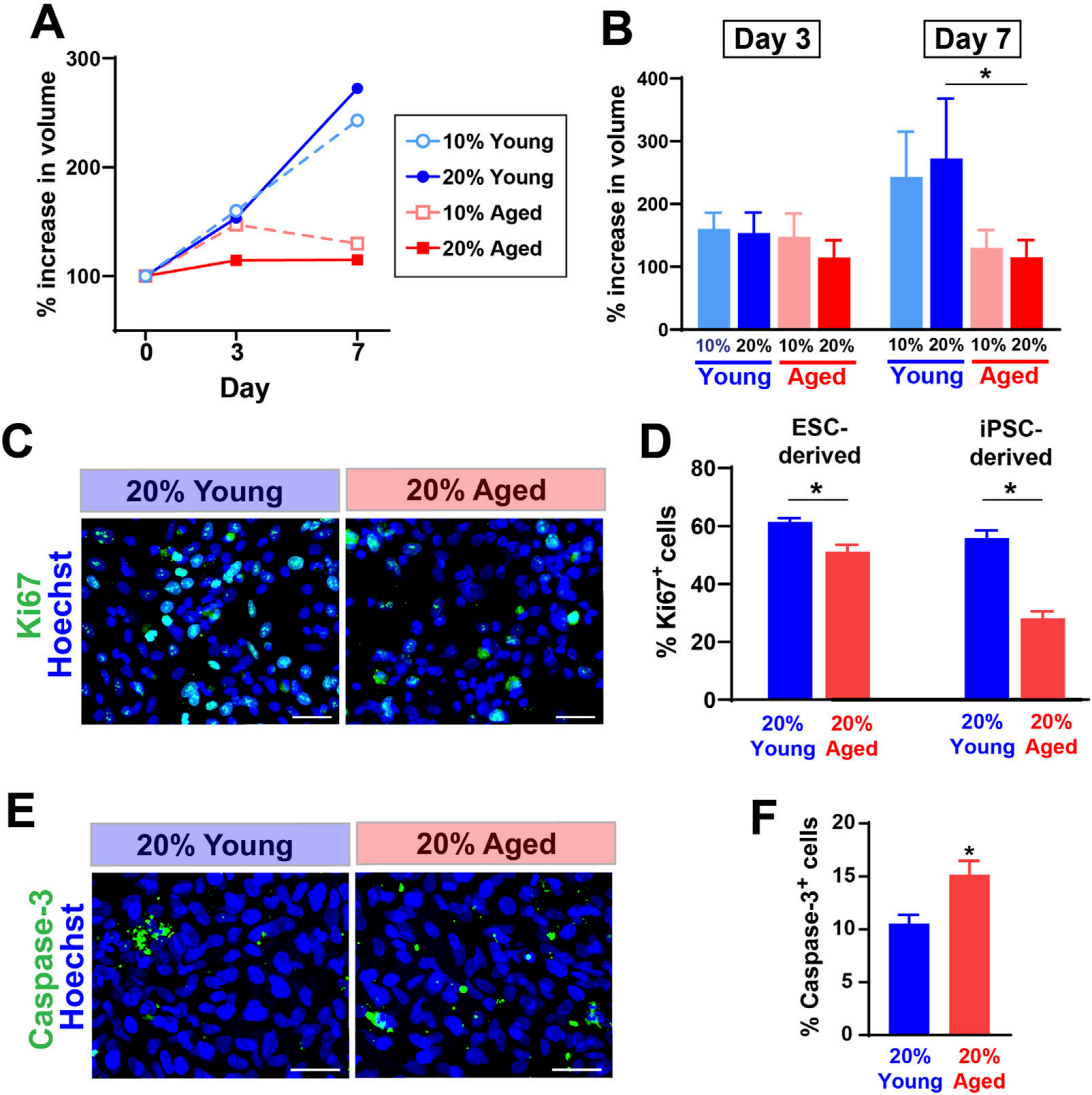
The supplementation of aged rat sera influenced cell proliferation and death in hPSC-derived myogenic progenitors. **(A)** % increase in sphere volume showing growth trends following serum supplementation in culture. **(B)** On Day 7, the sphere volume was significantly different between cells treated with young and aged rat sera. The graphs represent three independent experiments (n = 3 for each group). Statistical significance was calculated by two-way ANOVA (F_3,12_ = 3.68, *p* = 0.043) followed up by Tukey’s *post hoc* test. *q = 4.474, df = 12, *p* = 0.036. **(C)** Representative immunocytochemistry images of Ki67 expression after 2 days of serum supplementation. **(D)** The number of cells positive for proliferation marker Ki67 was significantly lower when 20% aged rat serum was supplemented in ESC- (t = 3.710, df = 10, *p* = 0.004) and iPSC-derived myogenic progenitors (t = 7.642, df = 10, *p* < 0.001). **(E, F)** In contrast, immunocytochemistry for an apoptotic marker Caspase-3 showed the increased cell death in iPSC-derived progenitor cells (t = 2.992, df = 10, *p* = 0.014). The results represent three independent experiments. Scale bar = 50 µm in **(C, E)** n = 6 each, *: *p* < 0.05 in **(D, F)**.

We first tested whether the treatment with aged rat sera would influence survival and expansion of hPSC-derived progenitor cells cultured as spherical aggregates (EZ spheres). EZ spheres were prepared from human ESC line WA09 (H9) and human iPSC line IMR-90. Individual spheres were transferred in each well of a 96-well plate and cultured in the expansion medium supplemented with two different concentrations (10% or 20%) of young or aged sera. These rat serum samples were originally collected and pooled from young (6–9 months old) or aged (30–32 months old) F344/BN male rats. The sphere volume perpendicular diameters of each sphere were measured to calculate the sphere volume. Our recent studies indicate the growth in sphere size represents positive expansion rate of human progenitor cells (Wright et al., 2006; Tey et al., 2022). By Day 7, the growth rate of spheres treated with young adult rat sera had surpassed that of spheres treated with aged rat sera (Figures 1A, B). There is a significant difference between cells treated with 20% young adult rat sera and 20% aged rat sera on Day 7 (Figure 1B).

To understand how the growth rate of spheres correlates with proliferation and cell death, we performed immunocytochemistry to detect the expression levels of a proliferation marker Ki67 and an apoptosis marker Caspase-3. After 2 days of rat sera supplementation, the dissociated cells from EZ spheres were plated on coverslips and fixed after a few hours. Our previous observations indicate that these acutely plated cells still remain as pre-differentiated progenitors in EZ spheres (Hosoyama et al., 2014; Jiwlawat et al., 2017). Ki67 expression was significantly lower in the cells treated with 20% aged rat sera when compared to the ones treated with 20% young adult rat sera (Figures 1C, D). Meanwhile, cells treated with aged rat sera significantly increased the expression of an apoptosis marker Caspase-3 (Figures 1E, F). Together, the supplementation of aged rat sera reduced the expansion rate of EZ sphere cells through reduced proliferation and increased apoptosis.

### 3.2 Aged rat sera reduced muscle differentiation capacity in hPSC-derived myogenic progenitors

**FIGURE 2:**
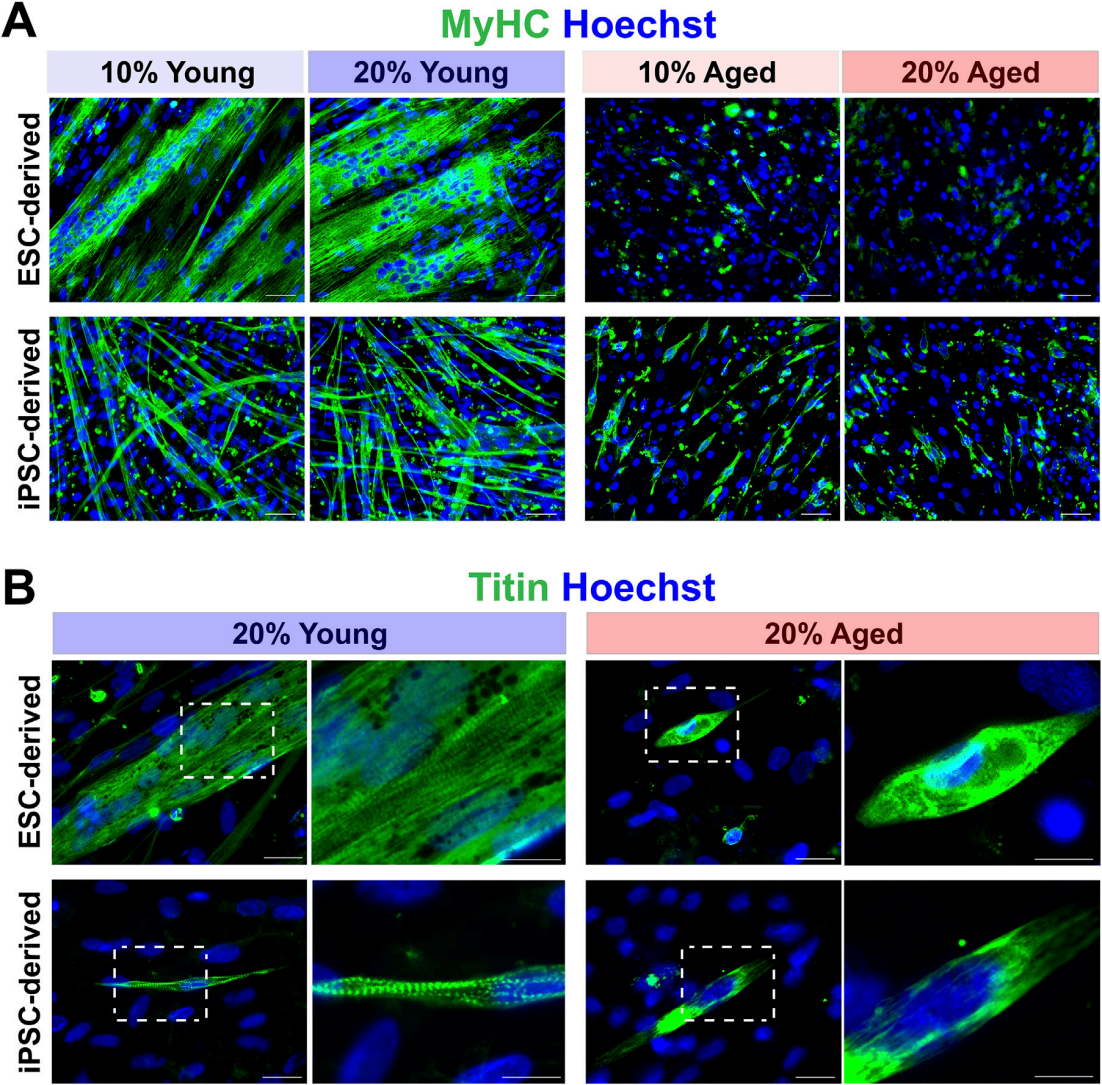
Effects of aged rat sera supplementation on myogenic differentiation capacity of hPSC-derived myogenic progenitors. **(A)** Representative images of immunolabeling with myosin heavy chain (MyHC). Cells supplemented with young rat sera formed long, thick, multinucleated MyHC^+^ myotubes. ESC-derived myogenic progenitors supplemented with aged rat sera barely formed any myotubes. Some short, single-nucleated myotubes were present in the of iPSC-derived differentiated cultures. Scale bar = 50 µm. **(B)** Striated patterns of sarcomere filament titin expression were clearly visible in myotubes formed by young rat sera-treated cells. In contrast, myotubes formed under aged rat sera supplementation contained undefined filaments with no sarcomere structure. Scale bar = 20 µm. Enlarged images scale bar = 10 µm.

We next determined whether the supplementation of aged rat sera would influence the ability of muscle differentiation and myotube formation from hPSC-derived myogenic progenitors. Dissociated EZ sphere cells were plated down on glass coverslips and then maintained in the serum-supplemented medium up to 14 days, which can induce terminal differentiation into myotubes. Four different conditions were tested for serum supplementation: 10% young adult rat sera, 20% young adult rat sera, 10% aged rat sera or 20% aged rat sera. Immunostaining for myosin heavy chain (MyHC) revealed that the myotubes differentiated from young adult rat sera-treated cells appeared longer, thicker, multinucleated, and highly fused (Figure 2A). Their myonuclei lined up along the major axis in a train-like orientation, while the myotubes displayed visible striated patterns when immunolabeled with a sarcomere protein, titin (Figure 2B).

**FIGURE 3:**
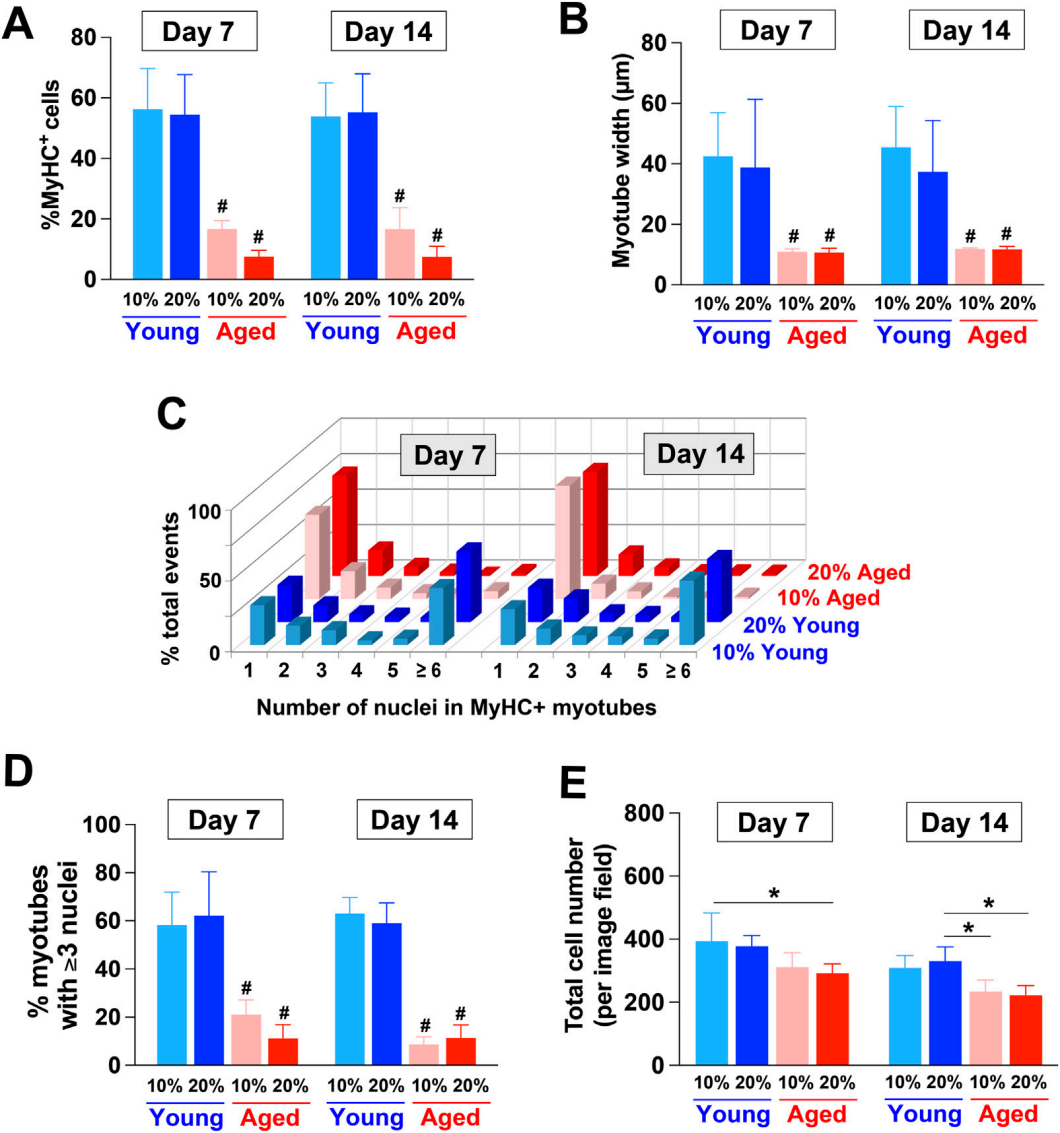
Quantitative characterization of myotubes formed by hPSC-derived myogenic progenitors supplemented with aged rat sera. **(A)** On Day 7 and 14, the supplementation of aged rat sera significantly reduced the proportion of MyHC^+^ cells when compared to young adult rat sera (2-way ANOVA, F_3,9_ = 9.743, *p* = 0.003). The width of formed myotubes (2-way ANOVA, F_3,9_ = 2.458, *p* = 0.129) **(B)** and the proportion of multinucleated myotubes with more than 3 nuclei **(C, D)** also showed similar trends (2-way ANOVA, F_3,9_ = 14.81, *p* < 0.001). **(E)** Additionally, the total number of cells was reduced in the cultures of aged rat sera-treated cells (2-way ANOVA, F_3,9_ = 3.801, *p* = 0.052). n = 4 for each group ^#^ *p* < 0.01 vs. young serum-treated groups *p < 0.05.

We also characterized the degrees of myotube formation by quantifying different parameters (Figure 3). When compared to young adult rat sera, the supplementation of aged rat sera significantly reduced the proportion of MyHC^+^ cells (Figure 3A), the width of myotubes formed (Figure 3B), and the proportion of multinucleated myotubes with more than 3 nuclei (*p* < 0.01) (Figures 3C, D). These results indicated impaired myotube formation, fusion, and maturation by aged sera supplementation. Additionally, the total number of cells in the image field was reduced in the cultures of aged rat sera-treated cells on Day 7 and 14 (*p* < 0.05; Figure 3E).

### 3.3 hPSC-derived myogenic progenitors supplemented with aged rat sera showed signs of cell cycle arrest upon induction to terminally differentiate

**FIGURE 4:**
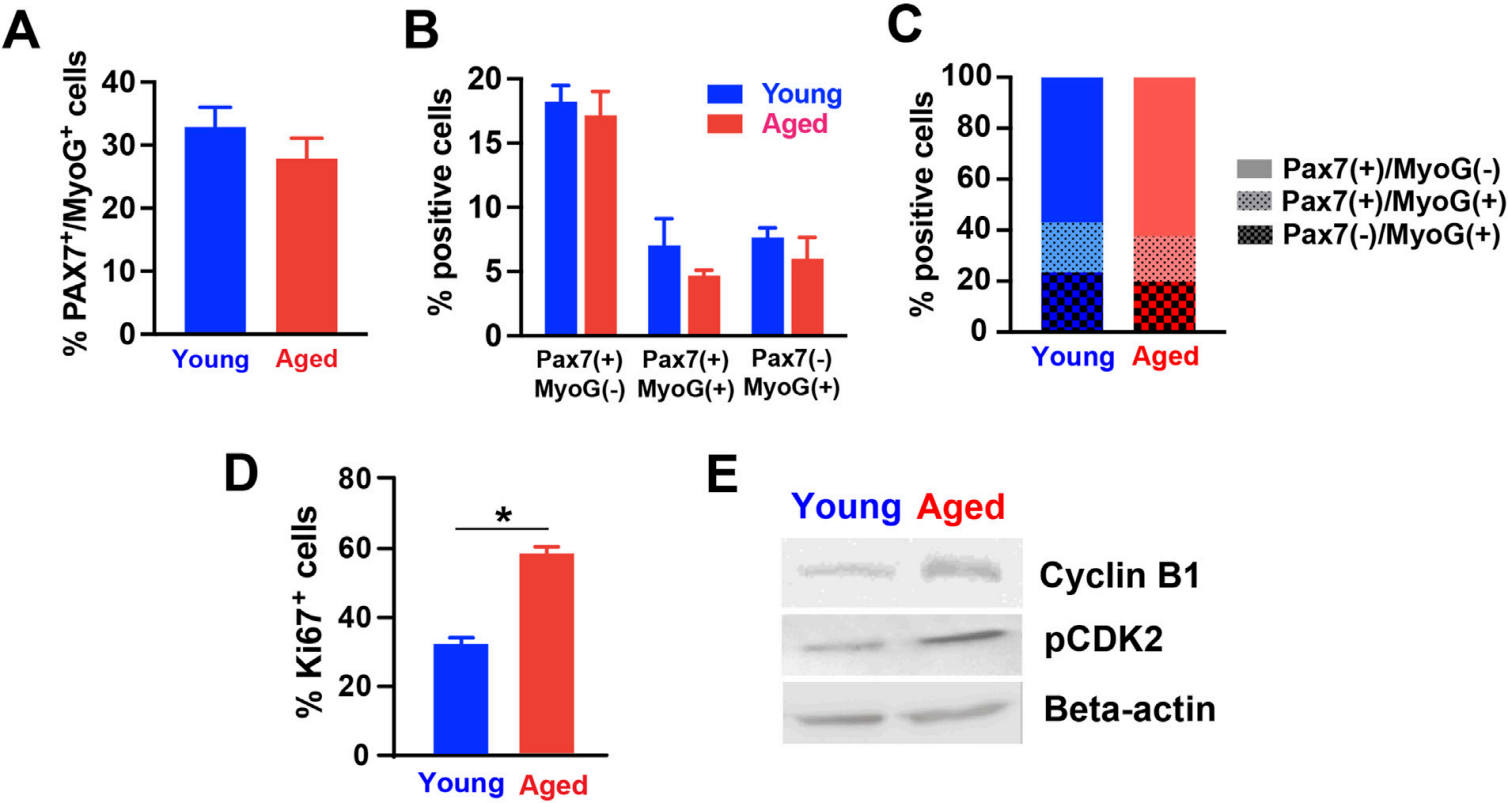
Expression levels of various markers in differentiated hPSC-derived myogenic progenitors treated with rat sera. The expression of Pax7 and Myogenin (MyoG) proteins were analyzed in the differentiated cells for 3 days with 20% young or aged rat sera. **(A)** The type of supplemented sera did not alter the percentage of total myogenic cells (expressing PAX7, MyoG, or both) (Student’s *t*-test, t = 1.115, df = 10, *p* = 0.291). There was no difference in the number **(B)** and proportions **(C)** of quiescent, activating and activated myogenic progenitors out of total cells (represented by cells expressing PAX7^+^/MyoG^−^, PAX7^+^/MyoG^+^ and PAX7^−^/MyoG^+^, respectively) (2-way ANOVA, F_1,30_ = 1.958, *p* = 0.172 for **(B)**; Student’s *t*-test, t < 0.001 df = 2, *p* > 0.999 for **(C)**. **(D)** On Day 7 of terminal differentiation, cells supplemented with aged rat sera showed an increased level of a cell proliferation marker Ki67 (t = 9.357, df = 10, *p* < 0.001; by unpaired two-tailed Student’s t-test). **(E)** Western blot analysis showed increased cyclin B1 and CDK 2 expression in hPSC-derived myogenic progenitors differentiated in 20% aged rat sera for 2 weeks. Beta-actin served as loading control. n = 6 for each group *p < 0.05.

We further examined the expression of different markers to characterize myogenic stages, cell proliferation, and cell cycle. For this analysis, we used the cultures of hPSC-derived myogenic progenitors supplemented in 20% young adult or aged rat sera. Firstly, co-staining with PAX7 (muscle progenitors) and myogenin (MyoG, committed myocytes) was performed to identify the stage of myogenic cells (Jiwlawat et al., 2017). At 2 days after induction of terminal differentiation, the percentage of total myogenic cells (i.e., cells expressing PAX7, MyoG, or both) was not altered by the type of supplemented sera (Figure 4A). Similarly, the proportions of quiescent, activating and activated myogenic progenitors out of total cells (represented by cells expressing PAX7^+^/MyoG^−^, PAX7^+^/MyoG^+^ and PAX7^−^/MyoG^+^, respectively) did not differ between groups (Figure 4B). These results imply that the reduced myotube formation in aged rat sera-treated cells was not caused by changes in cell types in the cultures, as commitment to the myogenic lineage remained unchanged. Similarly, the proportions of cells expressing PAX7^+^/MyoG^−^, PAX7^+^/MyoG^+^ and PAX7^−^/MyoG^+^ out of total myogenic cells were also not significantly different between groups (Figure 4C), suggesting that aged rat sera supplementation did not affect the activation of myogenic progenitors during progression of terminal differentiation.

We next determined how young adult and aged sera supplementation impacted cell proliferation and cell cycle. On Day 7 of terminal differentiation, cells supplemented with aged rat sera showed an increased level of a cell proliferation marker Ki67 (Figure 4D). This was an unexpected result when considering our earlier results in Figure 3E, because total cell number was reduced when aged rat sera were supplemented to the plated cells (Figure 3E). These observations led us to speculate the occurrence of aberrant cell cycle reentry or cell cycle arrest in aged rat sera-treated cells. To test this working hypothesis, we prepared protein lysates from sera-treated cells, performed Western blot, and determined the level of cell cycle markers cyclin B1 and CDK2. Both cyclin B1 and CDK2 proteins were detected more in the cells supplemented with aged sera when compared to young sera supplementation (Figure 4E).

### 3.4 Young adult rat sera could rescue the inhibitory effects of aged rat sera during terminal differentiation of hPSC-derived myogenic progenitors

**FIGURE 5:**
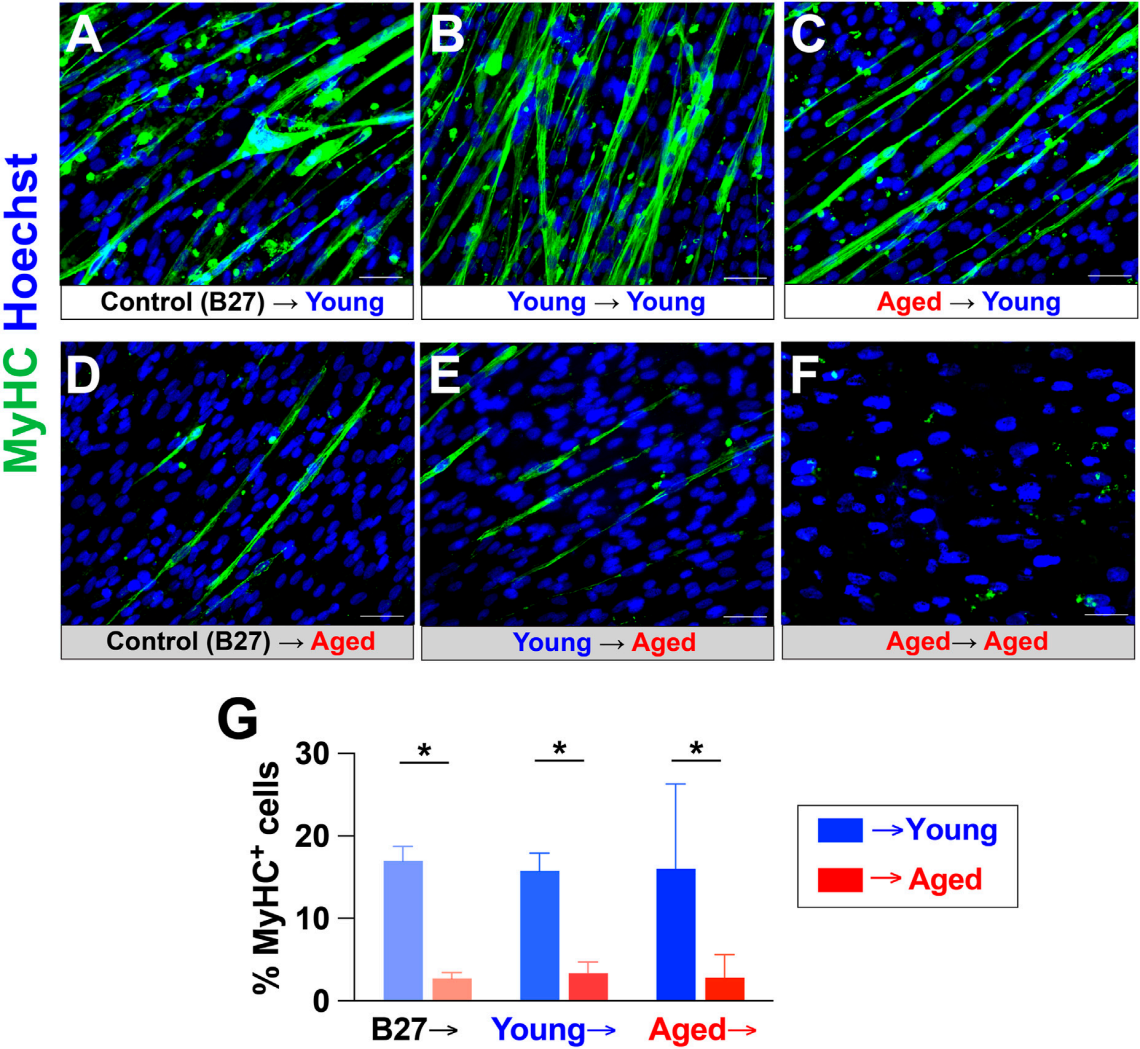
Young rat sera supplementation rescued the inhibitory effects of aged rat sera supplementation on myotube-forming ability of hPSC-derived myogenic progenitors. Myogenic progenitors were prepared as EZ spheres and plated on glass coverslips. The plated cells were first maintained for 2 weeks in 2% B27 supplement (control), 20% young rat sera, or 20% aged rat sera. The cultures were then switched to medium supplemented with either young or aged rat sera. MyHC immunocytochemistry revealed that young rat sera supplementation at the end of the 3-week culture increased myotube formation **(A–C)**, whereas aged rat sera supplementation in the final week yielded a low number of myotubes **(D–F)**. Notably, cells cultured in aged rat sera for 2 weeks were able to regain myotube-forming ability after being switched to treatment with young rat sera for 1 week **(C)**. In contrast, myotube formation and survival (as implied by reduced cell density) were vastly reduced in cells cultured in aged rat sera for 3 continuous weeks **(F)**. **(G)** Quantification of MyHC^+^ cells corresponding to Figure 4B. Error bars represent SEM from three independent experiments. Statistical significance was calculated by two-way ANOVA (F_1,14_ = 14.99, *p* = 0.002) followed by Tukey’s *post hoc* test *p < 0.05.

We next determined whether young adult rat sera could rescue the inhibitory effects of aged rat sera on myotube formation and maturation in hPSC-derived myogenic progenitors. Plated cells were first differentiated for 2 weeks in one of the following: 1) terminal differentiation medium (TDM; DMEM with 2% B27 serum-free supplement) as a control, 2) TDM additionally supplemented with 20% young adult rat sera, or 3) TDM supplemented with 20% aged rat sera. The cells were then cultured in new TDM with either 20% young adult rat sera or 20% aged rat sera for another week (Figure 5). Immunocytochemical analysis showed that 1-week exposure to young adult rat sera was able to rescue myotube-forming ability in the cells differentiated in aged sera-supplemented medium for 2 weeks (Figure 5C), whereas cells differentiated in aged rat sera for 3 weeks barely formed any myotubes (Figure 5F). Of note, regardless of the initial treatment type in the first 2 weeks, young adult sera supplementation in the final week commonly yielded the greater number of myotubes (Figures 5A–C), whereas the cells supplemented with aged rat sera in the final week had diminished myotube formation (Figures 5D–F). Specifically, the cells treated aged rat sera for the first 2 weeks increased the number of myotubes after being administered young rat sera for a subsequent week (Figure 5C). On the other hand, the administration of aged rat sera for 3 continuous weeks showed the lowest degree of myotube formation (Figure 5F). Image quantification also confirmed that the number of MyHC^+^ cells was significantly higher in cultures treated with young adult rat sera in the final week when compared to their counterparts treated with aged rat sera (*p* < 0.05; Figure 5G). These results indicate that supplementing hPSC-derived myogenic progenitors with young adult rat sera could revert detrimental effects of treatment with aged rat sera on myotube formation.

### 3.5 Profiling initial transcriptomic response of hPSC-derived myogenic progenitors exposed to young and aged serum

**FIGURE 6:**
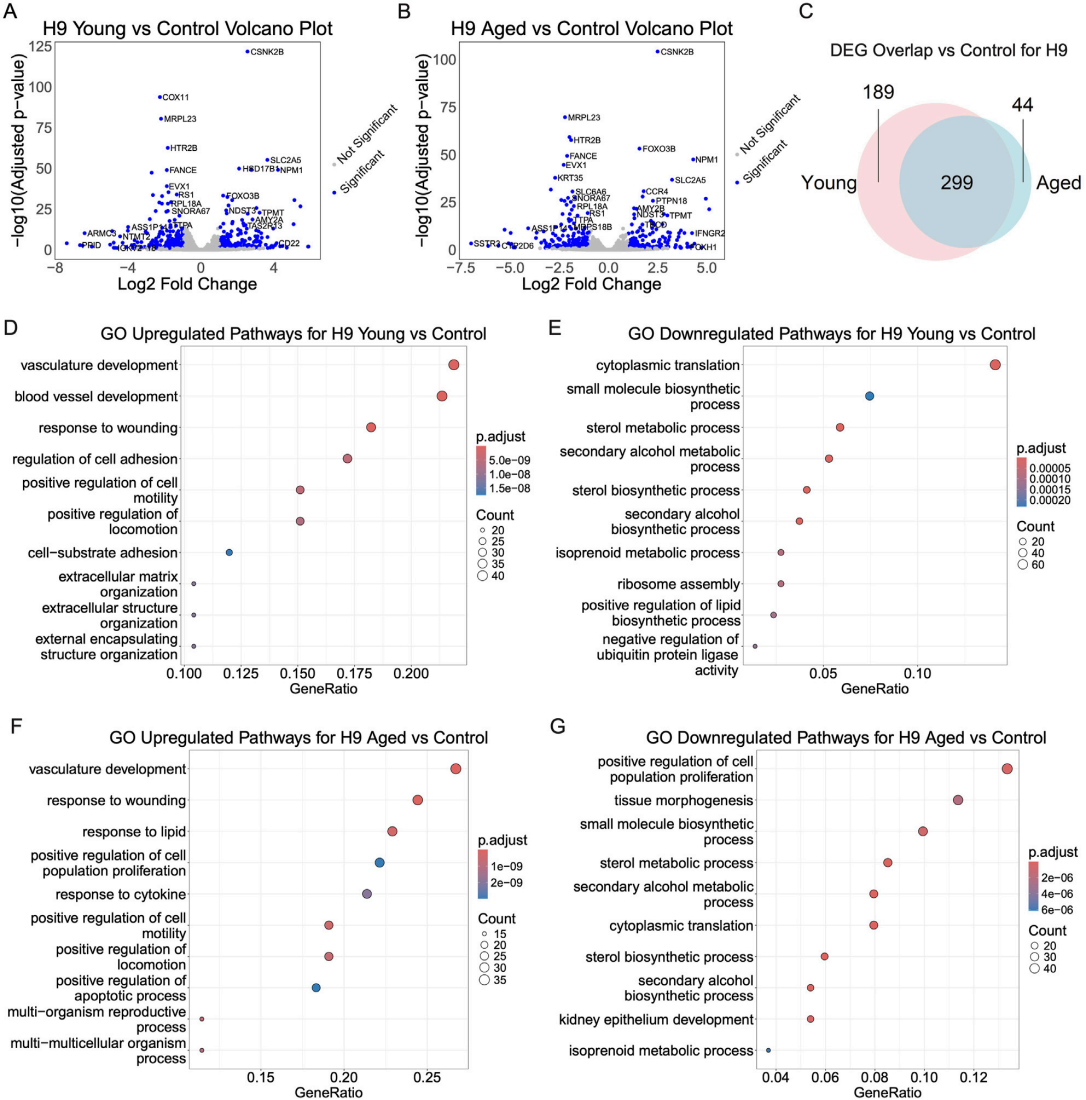
Transcriptomic changes of ESC-derived myogenic progenitors upon supplementation with aged, young, or no serum. To investigate transcriptomic changes for serum supplementation paradigms, EZ-spheres were supplemented with aged serum, young serum, or no serum. RNA for Bulk RNA barcoding and sequencing was extracted after 48 h and differential gene expression was performed with Benjamini–Hochberg-corrected Wald test adjusted *p*-value < 0.1 and |log2foldchange| > 1 as significance cutoffs. **(A, B)** The top differentially expressed genes depicted in the volcano plot from aged or young serum supplementation compared to controls. **(C)** Differentially expressed genes (DEGs) for young or aged serum supplemented samples compared to serum-free controls largely overlap with minor differences. **(D–G)** Gene ontology enrichment analysis of young or aged serum supplementation compared to serum-free controls reveals overlapping pathways of DEGs.

We sought to identify early transcriptomic alterations in hPSC-derived myogenic progenitors treated with aged serum, young serum, and serum-free medium for 48 h. Due to the limited availability of rat serum samples, BRB-seq was conducted with 2 replicates in each group from both iPSC and ESC-derived myogenic progenitors. PCA and sample clustering analysis using variant transformed stabilized gene counts shows clustering of replicates (Supplementary Figures S1A–D). ESC-derived myogenic progenitors had 343 total DEGs (157 upregulated; 186 downregulated) and 488 total DEGs (244 upregulated; 244 downregulated) for aged-serum and young-serum, respectively (Figures 6A, B). There were 738 total differentially expressed genes (DEGs) for aged-serum iPSC-derived myogenic progenitors (402 upregulated; 336 downregulated) and 701 for young-serum iPSC-derived myogenic progenitors (391 upregulated; 310 downregulated) (Supplementary Figures S2A, B). The differential gene expression data is included in Supplementary Table S1. The DEGs were largely overlapped throughout the progenitors from young and aged-serum supplemented iPSC and ESC-derived myogenic progenitors. When compared to controls, 299 of the DEGs overlapped for aged and young-serum supplemented ESC-derived myogenic progenitors and 536 DEGs overlapped for iPSC-derived myogenic progenitors (Figure 6C; Supplementary Figures S2C). A direct comparison between young and aged-serum revealed no DEGs for both iPSC and ESC-derived myogenic progenitors with a |log2foldchange| < 1. Notably, genes previously identified as being involved in skeletal muscle aging processes, such as *FOXO3*, *LDHA*, and *HBEGF* for ESC-derived myogenic progenitors (Nakata et al., 1996; Washington et al., 2014; Jing et al., 2023) and *AKR1B* for iPSC-derived myogenic progenitors (Sataranatarajan et al., 2020), had an adjusted *p*-value less than 0.05 but |log2foldchange| < 1 (Supplementary Table S1).

Lastly, a gene ontology enrichment analysis was performed to identify overlapping differentially expressed pathways. The top upregulated pathways for ESC-derived myogenic progenitors greatly overlapped for samples exposed to young and aged serum. Top overlapping upregulated pathways include vasculature development, positive regulation of cell motility, and positive regulation of locomotion (Figures 6D, F). Of note, regulation of apoptotic processes and response to lipids were upregulated in just cells exposed to aged serum. Top overlapping downregulated pathways included cytoplasmic translation, small molecule biosynthetic process, sterol metabolic process, secondary alcohol metabolic process, sterol biosynthetic process, secondary alcohol biosynthetic process, and isoprenoid metabolic process (Figures 6E, G).

iPSC-derived myogenic progenitors showed similar trends in the pathway analysis results, with samples exposed to young and aged serum both showing upregulation in regulation of cell-cell adhesion, response to wounding, angiogenesis, response to xenobiotic stimulus, and positive regulation of vasculature development (Supplementary Figures S2D, F). Top overlapping downregulated pathways include skeletal system development, actin filament-based process, tissue morphogenesis, connective tissue development, cartilage development, and cardiac chamber morphogenesis (Supplementary Figures S2E, G).

## 4 Discussion

In this study, we found that the supplementation of aged rat sera impaired cell proliferation of undifferentiated progenitors in hPSC-derived EZ spheres. The reduced capacity of cell proliferation was represented by the decreased expansion rate of EZ spheres following aged sera supplementation. Notably, similar inhibitory effects on sphere growth were observed when aged human plasma samples were supplemented. Predictably, the reduced expansion rate of aged sera-treated spheres was a result of decreased cell division and increased cell death. These results contradict an earlier report using human myoblasts treated with 15% human serum from young and aged donors (represented by Ki67 expression) (George et al., 2010). Since human serum samples were supplemented for only 46 h in the previous study, it may not be long enough for the cells to react to the age-related difference in serum composition, especially after being conditioned with prolonged exposure (several passages) to medium containing fetal calf serum.

Consistent with previous studies using human primary cells (Carlson et al., 2009; Barberi et al., 2013; Romagnoli et al., 2021), we also observed an impairment in differentiation capacity following the supplementation of aged blood liquid components. Specifically, the impairment in differentiation capacity was represented by the decrease in myotube formation, myotube diameter, the number of nuclei per myotube (fusion index), and the visible alignment of sarcomere structure in differentiated cultures of the treated cells. The effects of aged sera on human myotube maturation (sarcomere formation) had not been shown in previous studies using muscle-derived cells, likely because primary cultures tend to be vulnerable to stressors and difficult to maintain for long durations. In this study, we demonstrated that hPSC-derived myogenic progenitors were able to withstand supplementation of 20% rat sera for 3 weeks of terminal differentiation. The high concentration of aged sera used in our study may explain the discrepancies with an earlier study that reported no difference in myogenic fusion index in myoblasts treated with 2% human serum from young and aged donors (George et al., 2010). As an additional note, we should acknowledge that the parameters we used to characterize the degree of cell differentiation may have some limitations for accurate quantification. For instance, thickly fused myotubes in cultures conditioned with young rat sera posed a challenge in determining what should be counted as one myotube.

We observed an unexpected increase of Ki67 expression in aged rat sera-treated cells at 7 days post-terminal differentiation, which did not correlate with either increased cell density or increased myotube yield (Figure 4G). This led us to hypothesize that impaired differentiation in aged rat sera-treated cells was caused by disrupted cell cycle progression. The Western blot results showing the elevation of cyclin B1 and CDK2 expression (Figure 4E) may further corroborate our hypothesis. Indeed, emerging evidence revealed that aberrant cell cycle initiation in differentiated mature muscles and neurons results in detrimental consequences following cell cycle checkpoints’ disarray, altered signaling cascades, and oxidative stress (Latella et al., 2001; Sacco et al., 2002; Pajalunga et al., 2010; Sharma et al., 2017; Pajalunga and Crescenzi, 2021). Aberrant cell cycle reentry or cell cycle arrest was reflected by increased expression levels of Ki67, proliferating cell nuclear antigen, cyclin E and cyclin D1 in the muscle samples from the patients with inclusion body myositis and polymyositis, as well as the brain samples from Alzheimer’s disease patients (Kwon et al., 2014). Our findings pointed towards this cell cycle abnormality as a strong possibility, although additional studies are still required to prove this. As resident myogenic progenitors in skeletal muscle, satellite cells have been known to initiate both self-renewal and differentiation through cell cycle activation (Latella et al., 2017; Kitzmann et al., 1998; Rao et al., 2016; Mohan and Asakura, 2017; Konagaya et al., 2020; De Luca et al., 2013; Heron-Milhavet et al., 2013; Subramaniam et al., 2014; Montesano et al., 2013; Zhang et al., 1999). Taken together with these previous observations, our current hypothesis is that age-specific changes in serum factors (most likely the altered balance of mitogenic and differentiative factors) send cues to the cells that trigger undesired cell cycle initiation. Myotube formation and maturation was hence impaired, as these dysregulated cell cycle events forced differentiating or differentiated muscle cells to enter a disturbed and vulnerable state. Unable to complete or exit the cell cycle, these cells could not progress towards terminal differentiation and eventually die.

In our culture preparation, the inhibitory effects of aged sera on myotube formation could be rescued by the subsequent supplementation of young rat sera in culture (Figure 5). These results support the idea that non-cell-autonomous factors in young rat sera may restore a youthful environment in the culture condition influenced by aged serum supplementation. Not limited to skeletal muscle tissues, it has been suggested that sustained exposure to a young systemic environment possibly rejuvenates aged organisms and promotes cellular function (Conboy et al., 2005; Mehdipour et al., 2020; Mehdipour et al., 2021). *In vivo* studies using heterochronic blood sharing, by parabiosis or direct plasma infusion, could rejuvenate old tissues while aging young tissues (Kamran et al., 2013; Yousefzadeh et al., 2022; Ansere et al., 2023). In contrast, other recent studies reported that systemic induction of senescence was induced in young mice after single heterochronic blood exchange (Jeon et al., 2022). Possible contributions of specific extrinsic factors to muscle rejuvenation and aging have n’t fully been ruled out yet, but some potential molecules have been proposed to explain the mechanism. Interestingly, a recent study introduced the involvement of circulating extracellular vesicles to the effects by serum on muscle progenitor cells and function (Sahu et al., 2021). Like our current approach, *in vitro* cell culture models have considerable advantages because it can simplify the intrinsic complexity of tissues for understanding extrinsic contributions of circulating factors in systemic aging (Veronesi et al., 2023). As an additional strength, human iPSCs can offer additional variations of cellular models to study cellular senescence and muscle aging under healthy and pathological conditions (Hosoyama et al., 2012; Khodabukus et al., 2018). Together, further studies would be valuable to identify specific factors and mechanisms responsible for the effects by serum supplementation in our culture setup. For instance, additional transcriptome profiling may be beneficial to identify any changes of gene expression following the subsequent supplementation of young rat sera.

A transcriptomic analysis looking at the initial response of hPSC-derived myogenic progenitors revealed that sera supplementation for 48 h leads to upregulation of genes involved in pathways such as wound repair, inflammation, cell adhesion, and angiogenesis with downregulation in pathways related to metabolic processes and tissue morphogenesis, but whether the serum is from young or aged rats had no significant impact on the transcriptome. A possible explanation for the absence of significant changes between young and aged serum-supplemented samples could be the short exposure time of 48 h. We sought to identify early transcriptomic changes induced by serum supplementation, but the changes may be more pronounced later in the differentiation process. This result would be consistent with previous studies demonstrating that aged and young serum-supplemented samples showed little difference after 46 h (George et al., 2010). Although no significantly large transcriptomic changes were identified, there were a few genes with adjusted *p*-values less than 0.05 but only a very mild log2foldchange. Included in these set of genes are *FOXO3*, *LDHA*, *HBEGF*, and *AKR1B* which have been shown to a play key role in age-related muscle maintenance and myogenesis (Nakata et al., 1996; Washington et al., 2014; Sataranatarajan et al., 2020; Jing et al., 2023). Of these genes with decreased expression, *FOXO3* is of particular interest because it is known that decreased *FOXO3* expression has been identified in aged primate and human skeletal muscle and contributes to human myotube senescence (Leger et al., 2008; Dalle and Koppo, 2021; Jing et al., 2023). Further, *FOXO3* plays an important role in maintenance of muscle stem cell populations, promoting their self-renewal and preventing their premature differentiation (Gopinath et al., 2014). Taken together with our morphologic evolutions, our findings indicate that non-cell-autonomous factors in serum may contribute to an early decrease in *FOXO3* which ultimately drives aberrant myotube homeostasis and impaired myogenic differentiation. Which factors and whether these factors directly or indirectly affect *FOXO3* mRNA expression is yet to be explored.

When compared to controls that are not serum supplemented, there are minor differences in the DEGs between aged and young-serum supplemented samples. Investigation of pathways enriched in aged serum-supplemented samples, but not young serum-supplemented samples, reveal that hPSC-derived myogenic progenitors show an upregulation of apoptotic pathways. This finding is consistent with our earlier analysis of an apoptotic marker Caspase-3 in hPSC-derived EZ spheres (Figures 1E, F). Interestingly, response to lipids was upregulated in both iPSC and ESC-derived myogenic progenitors supplemented with aged serum. In rodents (Eum et al., 2020) and in human (Johnson and Stolzing, 2019; Lin et al., 2024), blood lipid levels tend to increase with age. Interestingly, *FOXO* transcription factors, such as *FOXO3* described above, can drive a shift towards the use of lipids as an energy substrate as opposed to glucose (Canto et al., 2009). Our transcriptomic data also shows a decreased expression of genes associated with myogenic homeostasis that are involved in carbohydrate metabolism, *LDHA*, *HBEGF*, and *AKR1B* (Nakata et al., 1996; Washington et al., 2014; Sataranatarajan et al., 2020). With around two-thirds of energy at rest coming from lipid oxidation, lipid entry, and metabolism in skeletal muscle fibers, lipid metabolism is crucial for energy production; in aging changes in fatty acid metabolism lead to lipid accumulation in muscle cells, which contributes to insulin resistance and ceramide buildup (Al Saedi et al., 2022). Insulin resistance and ceramide buildup is a known driver of muscle cell apoptosis, a phenomenon experimentally observed in this study (Turpin et al., 2006). Our model highlights a potential mechanism in which rising lipid levels in blood and a shift towards lipid metabolism with age can contribute to muscle cell apoptosis. Understanding the influence of circulating factors in aged blood on lipid metabolism may yield useful insights into the mechanisms by which these factors lead to skeletal muscle decline with age.

In summary, findings from this study imply that the supplementation of young and aged rat serum samples differently promoted cellular and transcriptomic changes in hPSC-derived myogenic progenitors. Our *in vitro* model cannot completely recapitulate the complex *in vivo* milieu of age-associated skeletal muscle pathophysiology. However, it has experimental advantages that enable us to explore inaccessible biological processes of such aberrancy in a controlled setting. While this preliminary study was intended to use myogenic progenitors derived from normal hPSCs, the approach is also adaptable to use iPSCs or serum from patients of other neuromuscular diseases for studying other pathological or age-associated muscle disorders.

## Data Availability

The data presented in the study are deposited in the GEO repository, accession number GSE278717.
