# Supplementary material for: Cellular and transcriptomic changes by the supplementation of aged rat serum in human pluripotent stem cell-derived myogenic progenitors
Source: Front Cell Dev Biol. 2024 Oct 15;12:1481491. doi: 10.3389/fcell.2024.1481491 (PMC11518775; doi:10.3389/fcell.2024.1481491)
Supplement: Supplementary file 2 [file DataSheet1.DOCX]

Supplementary Material

## Supplementary Figures

**Supplementary Figure S1: Principal component and sample clustering analysis for hPSC-derived myogenic progenitor cells exposed to different serum supplementation conditions**. (**A**) ESC-derived myogenic progenitor principal component analysis based on variance stabilized transformed counts. Each dot represents a different sample and is color coated by condition. Axes represent principal components (PCs) explaining the highest variance in the data. (**B**) ESC-derived myogenic progenitors are clustered using a hierarchical clustering method. Darker blue indicates lower sample-to-sample distance of the transpose of the transformed count matrices. (**C-D**) Principal component analysis and sample clustering analysis, as described above, for iPSC-derived myogenic progenitor cells.


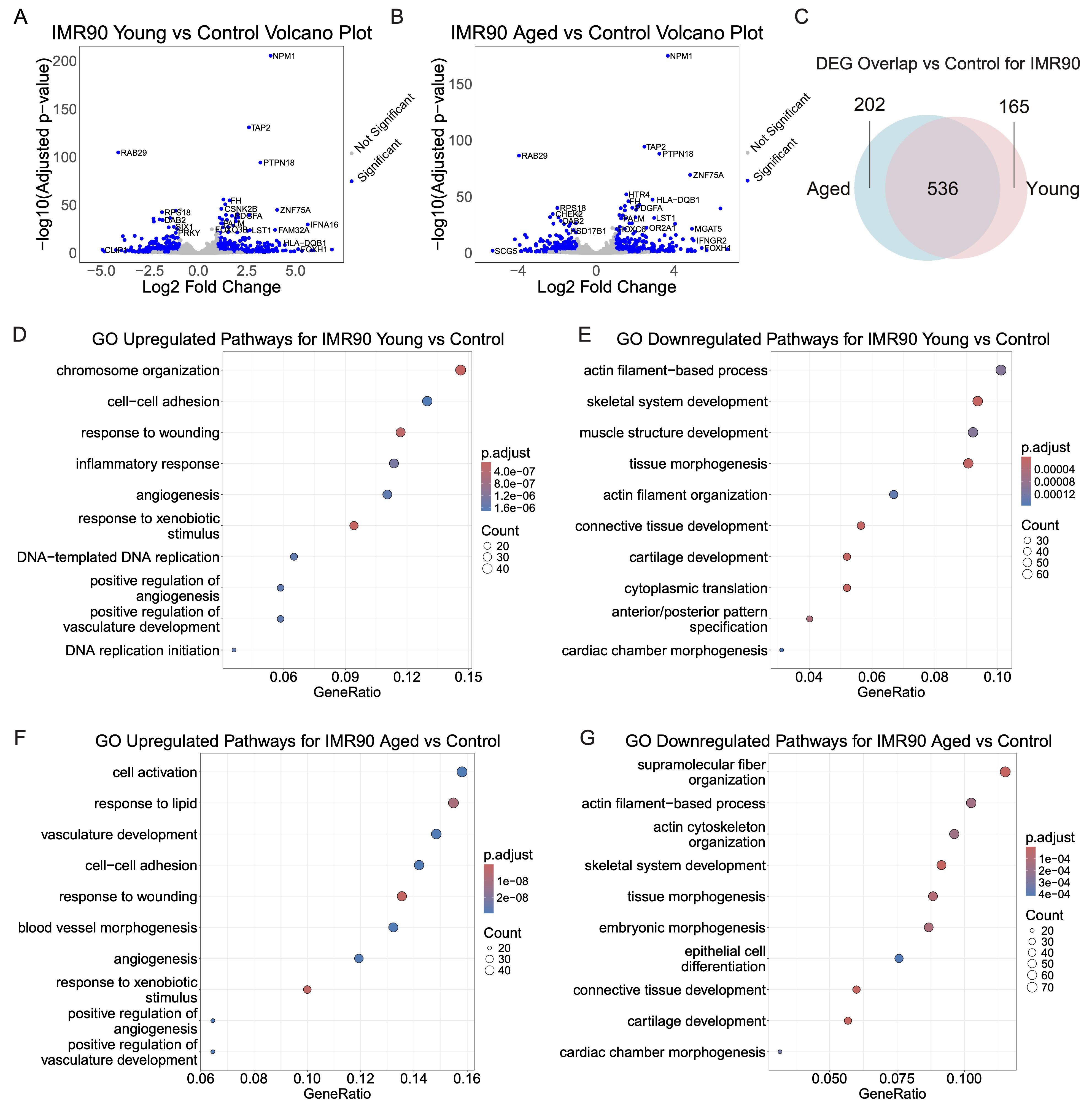


**Supplementary Figure S2. Transcriptomic changes of iPSC-derived myogenic progenitors upon supplementation with aged, young, or no serum.** To investigate transcriptomic changes for serum supplementation paradigms, EZ-spheres were supplemented with aged serum, young serum, or no serum. RNA for Bulk RNA barcoding and sequencing was extracted after 48 hours and differential gene expression was performed with Benjamini–Hochberg-corrected Wald test adjusted p value < 0.1 and |log2foldchange| > 1 as significance cutoffs. (**A-B**) The top differentially expressed genes depicted in the volcano plot from aged or young serum supplementation compared to controls. (**C**) Differentially expressed genes (DEGs) for young or aged serum supplemented samples compared to serum-free controls largely overlap with minor differences. (**D-G**) Gene ontology enrichment analysis of young or aged serum supplementation compared to serum-free controls reveals overlapping pathways of DEGs.
